# Surface Properties of Wild-Type *Rhizobium leguminosarum* bv. *trifolii* Strain 24.2 and Its Derivatives with Different Extracellular Polysaccharide Content

**DOI:** 10.1371/journal.pone.0165080

**Published:** 2016-10-19

**Authors:** Jolanta Cieśla, Magdalena Kopycińska, Małgorzata Łukowska, Andrzej Bieganowski, Monika Janczarek

**Affiliations:** 1 Department of Genetics and Microbiology, Maria Curie-Skłodowska University, Akademicka 19, 20–033, Lublin, Poland; 2 Institute of Agrophysics, Polish Academy of Sciences, Doświadczalna 4, 20–290, Lublin, Poland; Estacion Experimental del Zaidin—CSIC, SPAIN

## Abstract

*Rhizobium leguminosarum* bv. *trifolii* is a soil bacterium able to establish symbiosis with agriculturally important legumes, i.e., clover plants (*Trifolium* spp.). Cell surface properties of rhizobia play an essential role in their interaction with both biotic and abiotic surfaces. Physicochemical properties of bacterial cells are underpinned by the chemical composition of their envelope surrounding the cells, and depend on various environmental conditions. In this study, we performed a comprehensive characterization of cell surface properties of a wild-type *R*. *leguminosarum* bv. *trifolii* strain 24.2 and its derivatives producing various levels of exopolysaccharide (EPS), namely, *pssA* mutant Rt5819 deficient in EPS synthesis, *rosR* mutant Rt2472 producing diminished amounts of this polysaccharide, and two EPS-overproducing strains, Rt24.2(pBA1) and Rt24.2(pBR1), under different growth conditions (medium type, bacterial culture age, cell viability, and pH). We established that EPS plays an essential role in the electrophoretic mobility of rhizobial cells, and that higher amounts of EPS produced resulted in greater negative electrophoretic mobility and higher acidity (lower *pK*_*app*,*av*_) of the bacterial cell surface. From the tested strains, the electrophoretic mobility was lowest in EPS-deficient *pssA* mutant. Moreover, EPS produced by rhizobial strains resulted not only in an increase of negative surface charge but also in increased hydrophobicity of bacterial cell surface. This was determined by measurements of water contact angle, surface free energy, and free energy of bacterial surface–water–bacterial surface interaction. Electrophoretic mobility of the studied strains was also affected by the structure of the bacterial population (i.e., live/dead cell ratio), medium composition (ionic strength and mono- and divalent cation concentrations), and pH.

## Introduction

*Rhizobium leguminosarum* bv. *trifolii* is a soil gram-negative α-proteobacterium that establishes symbiosis with agriculturally important legumes, clovers (*Trifolium* spp.), under nitrogen-limited conditions. This type of plant–microbe interaction is essential for the functioning of the biosphere, since it provides nutrients to plants, making them independent of the external input from nitrogen fertilizers [1]. The establishment of symbiosis is a complex process that involves an exchange of several signal molecules, among which plant flavonoids and bacterial lipochito-oligosaccharides, also called Nod factors, are best characterized [2]. As a result of this interaction, nodules–specialized organs on the roots of a compatible host plant, are formed. Reduction of atmospheric dinitrogen to ammonia is conducted inside the nodules by bacteria differentiated into bacteroids.

In the absence of their host plants, rhizobia must often survive long periods of time in the soil as free-living organisms. During that time, they are exposed to several environmental factors such as nutrient limitation, pH, salinity, drought, temperature changes, and oxidative stress [3–7]. Rhizobia have developed many strategies that allow them to survive in these detrimental conditions. Acidic exopolysaccharide (EPS) produced in large amounts by *R*. *leguminosarum* forms the outermost layer surrounding bacterial cells and plays an essential role in protecting the cells against desiccation and other stress factors [8]. This polysaccharide is also crucial for the attachment and biofilm formation on both abiotic surfaces and plant roots, as well as the establishment of effective symbiosis, especially with legumes that form indeterminate-type nodules (e.g., clover, pea, vetch, alfalfa) [2,9–11]. Strains of *R*. *leguminosarum* bvs. *trifolii* and *viciae* and *Sinorhizobium meliloti* that do not produce EPS induce only partially infected or even uninfected nodules on their compatible host plant roots, ineffective in nitrogen fixation [9,12–15]. However, the role of EPS in symbiosis seems to be more complex and dependent on the type of the legume host. For example, EPS of *S*. *fredii* HH103 is not required for nodulation of *Glycyrhiza uralensis* that also forms indeterminate-type nodules [16,17].

It was recently established that the succinyl groups in *S*. *meliloti* succinoglycan are critical for successful host root invasion, whereas succinoglycan chain length is much less important for its role in symbiosis [18]. *S*. *meliloti exoH* mutant, which produces large amounts of succinoglycan but cannot succinylate it, is unable to invade the host roots [18]. Recent data indicate that EPS plays an essential role in symbiosis also in some legumes forming determinate nodules (e.g., *Lotus japonicus* and *L*. *corniculatus*) [19–21]. The role of rhizobial EPS as a signal molecule in the early stages of symbiosis was confirmed, and a receptor-like kinase EPR3 was identified in *L*. *japonicus* that was responsible for the recognition and binding of compatible EPS produced by *Mesorhizobium loti* [19]. A conditional requirement for EPS was identified in this symbiotic model, since *M*. *loti* R7A mutant strains affected in the early EPS biosynthetic steps (e.g., *exoB* mutant) induced with a delay nitrogen-fixing nodules on *L*. *corniculatus* and *L*. *japonicus* ‘Gifu’, whereas mutants impaired in the mid or late biosynthesis steps (e.g., *exoU* mutant) induced uninfected primordia on these host plants [20]. Furthermore, the structure of EPS produced by R7A and its mutants was characterized [21]. Wild-type low-molecular mass fraction (monomeric octasaccharide with three acetyl groups) was bound by the EPR3 receptor and elicited a positive symbiotic response of the host plant, in contrast with low-molecular mass fraction of *exoU* mutant (pentasaccharide with reduced acetylation) that resulted in an improper plant response [21].

Also, the structure of EPS synthesized by *R*. *leguminosarum* has been determined in detail. This heteropolymer is composed of octasaccharide repeating units that contain d-glucose, d-glucuronic acid, and d-galactose residues in a molar ratio 5:2:1, and are additionally modified with pyruvyl and *O*-acetyl groups [22,23]. Two genes, *pssA* and *rosR*, play a key role in EPS biosynthesis in *R*. *leguminosarum* [24,25]. The former, *pssA*, encodes glucosyl-IP-transferase participating in the first step of EPS synthesis and a mutation in this gene totally abolishes production of this polysaccharide. The latter, *rosR*, encodes a regulatory protein involved in positive regulation of this process and its mutation results in a substantial decrease of EPS synthesis (~3-fold) [24]. Strains with mutations of either gene are more sensitive to oxidative stress, with impaired attachment and biofilm formation, and induce non-nitrogen-fixing nodules on clover plants [4,10]. In contrast, multiple *rosR* and *pssA* copies significantly increase EPS production, and enhance nodule occupancy and symbiosis of *R*. *leguminosarum* bv. *trifolii* with the host plant [24].

In addition, the composition of rhizobial envelope is very important for the adaptation to changing environmental conditions. The outer surface of *R*. *leguminosarum* cells contains various polysaccharides (PSs), such as lipopolysaccharide (LPS), capsular polysaccharide (CPS), neutral polysaccharide, and gel-forming polysaccharide [26]. Among these, LPS anchored in the bacterial outer membrane is the dominant component [27,28]. This PS plays an important role in bacterial desiccation tolerance, biofilm formation on the soil particles and plant root surfaces, as well as successful infection of host plants and adaptation to conditions inside the nodules [29,30]. CPSs are tightly associated with the bacterial surface and are neutral or acidic PSs with a structure very similar to or even identical with EPS [8].

Strain-dependent chemical composition of the cell envelope can influence bacterial physicochemical properties, such as surface electrical charge and hydrophobicity/hydrophilicity. These properties of the rhizobial envelope seem to play an important role in several physiological processes, including motility, cell growth, and division, attachment to abiotic and biotic surfaces, aggregation, and interaction between microorganisms and their environment [10,31–33]. These processes may be governed, to a certain degree, by the surface charge of bacterial cells as well as the electrostatic and hydrophobic interactions. In general, the net surface charge is negative in most bacteria, including rhizobia, and its magnitude can change depending on several factors, such as environmental pH, the surrounding medium, and cell viability [33–35]. Bacterial surface components contain several functional groups (e.g., phosphate, carboxylate, acetyl, and amino groups) that are ionized as a function of pH, thereby conferring electric charge to the cell periphery [36]. Thus, physicochemical parameters of the surface, such as the electric charge, are fundamentally important by influencing the overall cell polarity, conferring and maintaining the degree of surface hydrophilicity required for optimal cell function [34].

No comprehensive data concerning the surface properties of rhizobia and the influence of different factors, such as medium composition, pH, culture growth phase, and the level of produced EPS, on these bacterial properties are currently available. The aim of this work was the characterization of surface properties of wild-type *R*. *leguminosarum* bv. *trifolii* strain 24.2 and its derivatives differing with respect to the amounts of synthesized EPS, under different growth conditions. To the best of our knowledge, this is the first report describing rhizobial cell surface properties in detail using such approaches as measurements of electrophoretic mobility, water contact angle, surface free energy, and free energy of bacterial surface–water–bacterial surface interaction.

## Materials and Methods

### Bacterial Strains, Culture Conditions, and Sample Preparation

*R*. *leguminosarum* bv. *trifolii* wild-type strain 24.2 (Rt24.2) and its derivatives used in this study are listed in Table 1. Rt5819 and Rt2472 are mutant strains obtained via random mutagenesis, which contain a mini-Tn*5* transposon insertion in *pssA* and *rosR* genes, respectively. The presence of single copy of this transposon in the genomes of Rt5819 and Rt2472, the stability of these mutations, and restoration of all phenotypic and symbiotic defects in these strains by complementation of the mutations were previously demonstrated [24]. For experiments presented in this study, growth cultures of three independent colonies of each Rt24.2, Rt5819, Rt2472, Rt24.2(pBA1), and Rt24.2(pBR1) strain were used. To eliminate the possibility of additional mutations arising as a result of multiple bacterial passages, the bacteria were taken from their frozen stocks stored at -70°C. All these strains were grown in two energy-rich media, tryptone-yeast (TY) [37] and 79CA with 1% glycerol as a carbon source [38] at 28°C on a rotary shaker (160 rpm). The composition and selected characteristics of these media are presented in Table 2. Growth kinetics of the tested strains were determined in both TY and 79CA media by optical density (OD_600_) measurements of bacterial cultures after 0, 24, 48, 72, and 96 h. The experiments were repeated three times with three biological replicates for each strain analyzed.

**Table 1:** Bacterial strains used in this study.

Strains	Relevant characteristics	References
Rt24.2	Wild type, Rif^R^, Nx^R^	[24]
Rt2472	Rt24.2 derivative carrying a mini-Tn*5* transposon in the *rosR* gene, Km^R^	[24]
Rt5819	Rt24.2 derivative carrying a mini-Tn*5* transposon in the *pssA* gene, Km^R^	[24]
Rt24.2(pBA1)	Rt24.2 derivative carrying additional copies of the *pssA* gene on pBBR1MCS-2 vector, Km^R^	[24]
Rt24.2(pBR1)	Rt24.2 derivative carrying additional copies of the *rosR* gene on pBBR1MCS-2 vector, Km^R^	[24]

Rif^R^—rifampicin resistance, Km^R^—kanamycin resistance, Nx^R^–nalidix acid resistance

**Table 2:** Composition and characteristics of the bacterial media used in this study.

Component	Medium (concentration [g L^-1^])	
TY	79CA	
Bactro Trypton	5.0	-
Yeast Extract	3.0	1.0
Ca Casein hydrolyzate	-	1.0
Glycerol	-	10.0
Ca glycerophosphate	-	0.1
K_2_HPO_4_	-	0.5
NaCl	-	0.1
CaCl_2_ x 6H_2_O	1.3	-
MgSO_4_ x 7H_2_O	-	0.2
pH at 20°C	7.20	7.20
Ionic strength [mol dm^-3^]	0.018	0.027
Electrolytic conductivity at 20°C [mS cm^-1^]	2.40	2.46

To establish the effect of growth medium and growth time on surface properties of the studied strains, bacteria were grown in 5 mL of TY and 79CA for 24, 48, and 96 h. At these time points, OD_600_ of the cultures was measured, the cultures were equilibrated to OD_600_ = 0.4 using fresh liquid medium, and analyzed. The effect of pH on rhizobial surface properties was studied in TY and 79CA at different pH values ranging from 3 to 10 (equilibrated using NaOH or HCl). For these experiments, all strains were grown in the media for 24 h at 28°C. Then, OD_600_ was measured, bacterial suspensions were equilibrated to OD_600_ = 0.4, and analyzed. All experiments were repeated at least twice with three biological replicates for each strain and condition tested.

### Viability Determination of Rhizobial Cells

To evaluate the viability of bacterial cells grown in TY and 79CA at different pH values, the strains were cultured in 5 mL of these media for 24 h at 28°C with agitation. Cell viability assays were performed using two-component Bacterial Viability kit (LIVE/DEAD *BacLight* kit, Thermo Fisher Scientific, Waltham, MA, USA) as described previously [4]. Briefly, following OD_600_ measurements, bacterial cultures were centrifuged (10 min, 10,000 × *g*), washed twice with 0.9% NaCl and stained for 20 min in a mixture containing SYTO-9 (live cell stain) and propidium iodide (dead cell stain). Subsequently, bacterial suspensions were analyzed using Olympus SV1000 microscope, with 10 images acquired for each strain and condition tested. Live and dead cell numbers in cultures were calculated using a standard curve, and ImageJ 1.43e software (Wayne Rasband, NIH, MD, USA). The experiment was performed three times with three biological replicates for each strain, medium, and condition tested.

### EPS Isolation and Quantification

Isolation and quantification of EPS produced by rhizobial strains were performed according to a method described earlier [24]. Briefly, the strains were grown in 5 mL of 79CA and TY at 28°C for 24, 48, and 96 h. Then, OD_600_ of the cultures was measured and EPS was precipitated from supernatants obtained after centrifugation (20 min, 10,000 × *g*) with the addition of 96% cold ethanol (1:3 vol/vol). Subsequently, the samples were centrifuged and EPS pellets were air-dried, dissolved in deionized water, and analyzed for carbohydrate content according to Loewus [39]. Total sugar content was calculated as glucose equivalents per OD_600_ unit of the bacterial culture. The experiment was repeated three times with three biological replicates for each treatment and strain analyzed.

### Determination of the Electrophoretic Mobility (EM) of Rhizobial Strains Using Laser Doppler Velocimetry

To determine electrical properties of *R*. *leguminosarum* bv. *trifolii* strain surface, EM of bacterial cultures was measured using Zetasizer Nano ZS device (Malvern Instruments Ltd, Malvern, UK), as described previously [35]. For these experiments, cultures of all strains were grown in 79CA and TY media for different time periods (24, 48, and 96 h) at various pH conditions. Bacterial density of all cultures was equilibrated to OD_600_ = 0.4 using the appropriate medium. These experiments were performed three times with three biological replicates for each strain and treatment (nine independent measurements were done for each sample) at 20°C.

### Calculations of the Apparent Dissociation Constants (*pK*_*app*_) of Functional Surface Groups

To estimate the role of specific functional groups on the bacterial cell surface in the generation of electrical surface charge, potentiometric titration experiments were performed. Consumption of base solution during potentiometric titration corresponded to the neutralization of H^+^ ions released into the solution due to dissociation of surface acidic groups (BAH) of bacterial cells as well as acids (AH) in culture medium:

$$BAH+{OH}^{-}=BA^{-}+H_{2}O$$

$$AH+OH^{-}=A^{-}+H_{2}O.$$

The amount of base needed for neutralization of the surface charge of bacterial cells (N_B_) was calculated as: N_B_ = N_SUSP_ − N_M_, where N_SUSP_ was the amount of base consumed by the suspension and N_M_ was the amount of base consumed by the medium.

The distribution function of apparent dissociation constants (*pK*_*app*_) of the functional surface groups of bacterial cell envelopes was calculated using the following relationships [40,41]: *N*_*B*_(*pH*) = *Q*_*v*_(*pH*) = *Q*_*v*_(*pK*_*app*_) and *f*(*pK*_*app*_) = (1/*N*_*B*(*max*)_)(*dQ*_*v*_(*pK*_*app*_)/*dpK*_*app*_), where *N*_*B*_ was the amount of base used during titration, *Q*_*v*_ was the variable surface charge of bacterial cell, *f*(*pK*_*app*_) was the distribution function of apparent dissociation constants of surface functional groups, *N*_*B(max)*_ was the total amount of base used for titration, and *dQ*_*v*_(*pK*_*app*_)*/dpK*_*app*_ was the first derivative of *Q*_*v*_(*pK*_*app*_) function. Values of the distribution function of the apparent dissociation constants [*f(pK*_*app*_*)*] ranged from 0 to 1 and reflected the contribution of groups characterized by particular *pK*_*app*_ to the total surface electrical charge.

To evaluate the general acidic character of bacterial surface, the average apparent dissociation constant (*pK*_*app*,*av*_) was calculated:

$$pK_{app,av}=\sum_{i=1}^{n}pK_{{app}_{i}}f(pK_{{app}_{i}})$$

The lower the value of *pK*_*app*,*av*_ (as well as *pK*_*app*_, generally), the lower pH at which the surface functional groups dissociate (stronger acid), and the more acidic character of the surface.

Potentiometric titration was done using TitraLab TIM 965 Titration Workstation (Radiometer Analytical SAS, Lyon, France). The pH of 100-mL cultures (containing 0.4±0.08 g bacterial dry mass per L) and dispersing media was set to 3 by the addition of 1M HCl to avoid sample dilution. Titration was done in pH range from 3 to 10 using 0.1 M NaOH. The experiments were performed at 20°C, with three biological replicates for each strain.

### Contact Angle (CA) Measurements and Determination of the Surface Free Energy (SFE)

Hydrophilic/hydrophobic properties of bacterial cells were determined by a sessile drop technique. Bacteria from TY and 79CA cultures (pH 7.2) of similar optical densities (OD_600_≈0.4) were loaded onto membrane filters (cellulose membrane with 0.45 μm pore diameter, Whatman, GE Healthcare UK Ltd., Little Chalfont, UK) using negative pressure [42]. Then, the filters were air-dried for 40 min at room temperature and the CAs were measured using water, formamide, and diiodomethane. Measurements were done in triplicate for every biological repetition (three) for every strain at 20°C, using DSA100 microscope equipped with a goniometer and CCD camera (KRÜSS GmbH, Hamburg, Germany). A method described by van Oss and others [43] was used for the calculation of SFE and its components. Hydrophobicity/hydrophilicity of the bacterial cell was established using free energy value for the interaction between cell wall surfaces of two cells immersed in water (ΔG_BWB_) [44–46]:

$${\Delta G}_{BWB}=-2\left[{\left(\sqrt{\gamma_{B}^{LW}}-\sqrt{\gamma_{W}^{LW}}\right)}^{2}+2(\sqrt{\gamma_{B}^{+}\gamma_{B}^{-}}+\sqrt{\gamma_{W}^{+}\gamma_{W}^{-}}-\sqrt{\gamma_{B}^{+}\gamma_{W}^{-}}-\sqrt{\gamma_{W}^{+}\gamma_{B}^{-}})\right],$$

where $\gamma_{B}^{LW}$ and $\gamma_{W}^{LW}$ ($\gamma_{W}^{LW}$ = 21.8 mJ•m^-2^) were the apolar components of surface energy of bacteria (B) and water (W), respectively; and *γ*^+^ and *γ*^−^ were the electron-acceptor and electron-donor parameters of the polar (acid-base) component of surface energy ($\gamma_{W}^{+}{=\gamma }_{W}^{-}=25.5$ mJ•m^-2^).

### Statistical Analysis

The effect of growth media, rhizobial strain, and bacterial growth phase on the EM was analyzed using multi-way and one-way ANOVA and post-hoc HSD Tukey’s tests at α = 0.05 (Statistica v.10.0, StatSoft, Cracow, Poland). The results of all experiments are presented as arithmetic average with standard deviations.

## Results

### Growth and Survival of *R*. *leguminosarum* bv. *trifolii* Strains in 79CA and TY Media

**Fig 1:**
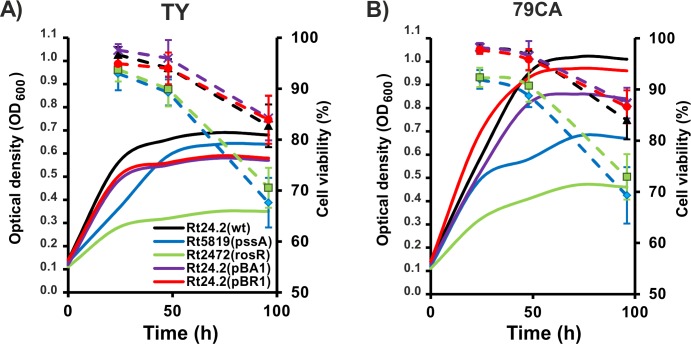
**Bacterial growth kinetics of the wild-type *R*. *leguminosarum* bv. *trifolii* strain 24.2 and its derivatives in TY (A) and 79CA (B) media.** Solid lines, OD_600_ of bacterial cultures; dashed lines, percentage (%) of live cells in bacterial populations.

First, growth kinetics of the wild-type *R*. *leguminosarum* bv. *trifolii* strain 24.2 and its derivatives were determined in two energy-rich media, TY and 79CA, over 96 h. In general, all the tested strains grew better in 79CA medium compared with TY medium, and the most rapid growth of these strains was observed during the first 48 h of the experiment (Fig 1). This was especially apparent for Rt24.2, Rt24.2(pBA1), and Rt24.2(pBR1) strains. In the case of TY medium (Fig 1A), the fastest growth was observed for Rt24.2, slightly slower for Rt24.2(pBA1) and Rt24.2(pBR1) strains, and the slowest for mutants Rt2472 and Rt5819. Strain Rt2472 (*rosR* mutant) was distinct on account of very slow growth under these conditions. A similar growth tendency was observed for all strains when 79CA medium was used (Fig 1B). Rt2472 and Rt5819 mutants grew more slowly than Rt24.2 and they did not achieve wild-type strain growth even after 96 h. These data indicated that the composition of 79CA medium favored the rhizobial growth more than that of TY, and *rosR* and *pssA* gene mutations negatively affected the growth of *R*. *leguminosarum* bv. *trifolii*.

Viability of *R*. *leguminosarum* bv. *trifolii* strains in 79CA and TY cultures at different time periods was determined using two fluorescent dyes specific for live and dead cells. In general, we determined that the numbers of live cells were very similar in both media for any individual strain analyzed, when the same time periods were compared (Fig 1). In TY medium, >90% of cells were alive in 24-h cultures of all the tested strains (Fig 1A). For Rt24.2, Rt24.2(pBA1), and Rt24.2(pBR1) strains, this number was maintained over the next 24 h and decreased to ~80% at 96 h. In the case of Rt2472 and Rt5819 strains, decrease of live cell numbers during a prolonged growth was more rapid, and 96-h cultures of these strains contained ~70% of viable cells. Similar viability profiles were obtained when these strains were grown in 79CA. Rt2472 and Rt5819 mutants exhibited lower viability than Rt24.2, Rt24.2(pBA1), and Rt24.2(pBR1) strains already after the first 24 h, and the viability of these bacteria decreased during the stationary phase of growth (72–96 h).

### EM of *R*. *leguminosarum* bv. *trifolii* Strains Grown in 79CA and TY Media, and the Influence of EPS

**Fig 2:**
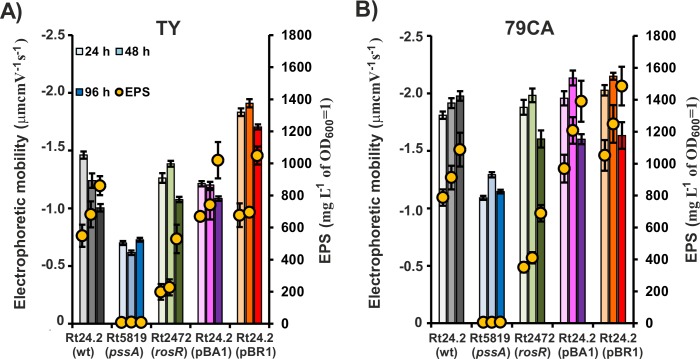
**Electrophoretic mobility and the amount of EPS produced by the wild-type *R*. *leguminosarum* bv. *trifolii* strain 24.2 and its derivatives in TY (A) and 79CA (B) media after 24, 48, and 96 h.** The intensity of bar color for individual strains increases with bacterial culture age.

Since rhizobia are well known to secrete large amounts of EPS, we next compared the amount of EPS produced by the wild-type strain Rt24.2 and its derivatives in 79CA vs. TY media, to establish whether this PS affects EM of rhizobial cells. To this end, the bacteria were grown in the two media and the amounts of EPS produced were determined after 24, 48, and 96 h. We determined that all strains, with the exception of Rt5819, synthesized significantly more EPS when they grew in 79CA medium compared with TY (when the same time periods were compared) (Fig 2). Rt5819 did not produce any EPS in either medium, in agreement with our previous results [24]. In the case of EPS-producing strains [Rt24.2, Rt24.2(pBA1), Rt24.2(pBR1), and Rt2472], the amounts of synthesized EPS increased with bacterial culture age and reached maximum after 96 h. From the analyzed strains, Rt24.2(pBA1) and Rt24.2(pBR1) produced more EPS, whereas Rt2472 synthesized significantly less EPS than the wild-type strain in 79CA as well as TY media (Fig 2). These results verified that the synthesis of EPS by *R*. *leguminosarum* bv. *trifolii* strains is more efficient when these bacteria are grown in 79CA medium.

EM is a parameter that characterizes surface properties of bacterial cells. Therefore, we determined EM of the wild-type strain Rt24.2 and its derivatives, and examined whether factors such as medium type, bacterial growth phase, and the amount of produced EPS influence this parameter. The electrolytic conductivity of TY and 79CA media was similar but the mineral/ionic composition of 79CA was more complex than TY, resulting in a higher ionic strength. However, the concentration of divalent cations was lower in 79CA compared with TY. We anticipated that we would be able to observe the effect of EPS despite the impact of the chemical composition of culture media on EM.

We observed that all the tested rhizobial strains exhibited negative EM values (Fig 2). Among them, Rt5819, which does not produce any EPS, was characterized by the lowest EM (absolute) value. EM value of mutant Rt2472 cells was moderately lower compared with the wild-type Rt24.2, and significantly higher than Rt5819 cells. EM values for Rt24.2(pBA1) and Rt24.2(pBR1) strains were the highest. These data indicated that the genetic modifications of Rt24.2 strain that affected EPS synthesis also significantly affected bacterial surface properties (F_0.05;2;240_ = 804.78, p<0.001).

Furthermore, EM values of individual strains were strongly correlated with culture age (F_0.05;2;240_ = 156.53, p<0.010). EM usually increased between 24 and 48 h, and then decreased (96 h), with the only exception noted for Rt5819 in TY. This positively correlated with increased EPS production by these strains during the first 48 h of growth. However, in 96-h cultures, decrease of the absolute EM values was observed for most tested strains in both media, although high amounts of EPS were produced. This suggested that another parameter (such as lower number of live cells) might have been additionally affecting rhizobial EM at this growth stage.

Multi-factor ANOVA analysis revealed that culture medium type also affected EM of the rhizobial cells (F_0.05;2;240_ = 2286.40, p<0.001). Generally, EM (absolute value) was higher for cells grown in 79CA than those grown in TY, when the same growth periods and individual strains were compared.

The obtained data indicated that factors such as bacterial culture age, growth medium type, and the amount of secreted EPS influence surface properties of *R*. *leguminosarum* bv. *trifolii* cells, measured as changes in EM values.

### Distribution of EM Values of Rhizobial Cells Grown in TY and 79CA Media

**Fig 3:**
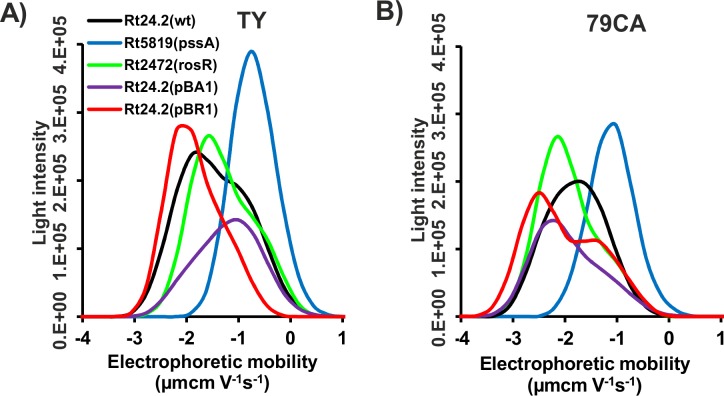
**Distribution of electrophoretic mobility of 24-h cultures of the wild-type *R*. *leguminosarum* bv. *trifolii* strain 24.2 and its derivatives at OD_600_≈0.4 in TY (A) and 79CA (B) media.**

To characterize surface properties of the studied strains in more detail, we analyzed EM distribution using the intensity of light scattered by the bacterial cells. This was done using 24-h TY and 79CA cultures at OD_600_ = 0.4 (Fig 3). The width and location of peaks for the individual strains reflected the variability of EM in analyzed cell suspensions. The location of peak maxima was dependent on the bacterial strain analyzed and the culture medium. The range of EM values for the wild-type strain Rt24.2 in TY was from -3.33 to 0.74, with the maximum at -1.88 μm•cm•V^-1^•s^-1^ (Fig 3A). The same range was found for Rt24.2(pBA1), albeit with a lower maximal value. Rt24.2(pBR1) and Rt2472 had a slightly shifted mobility range [Rt24.2(pBR1), from -3.35 to 0.14 μm•cm•V^-1^•s^-1^; Rt2472, from -3.05 to 0.74 μm•cm•V^-1^•s^-1^] in relation to that of Rt24.2, whereas their maximum values were very similar to that of Rt24.2. The greatest difference was observed for Rt5819. The mobility range of this strain was strongly shifted (from -2.20 to 1.00 μm•cm•V^-1^•s^-1^, with a maximum at -0.74 μm•cm•V^-1^•s^-1^) in comparison with the remaining strains. When 79CA rhizobial cultures were analyzed, similar peak locations for the individual strains were observed (Fig 3B), although the maximal mobility values for some of the tested strains [Rt24.2(pBR1), Rt2472, and Rt5819] were slightly lower than in TY. Rt24.2 strain showed very similar mobility distribution profile in both media. Also, in the case of 79CA cultures, Rt5819 cells showed a considerably altered mobility that was reflected by its range from -2.77 to 0.72 μm•cm•V^-1^•s^-1^ with a maximum at -1.02 μm•cm•V^-1^•s^-1^. These data suggested that the surface properties of EPS-non-producing strain Rt5819, as characterized by EM distribution, essentially differed from those of EPS-producing strains.

### Characterization of Functional Groups on the Rhizobial Cell Surface

**Fig 4:**
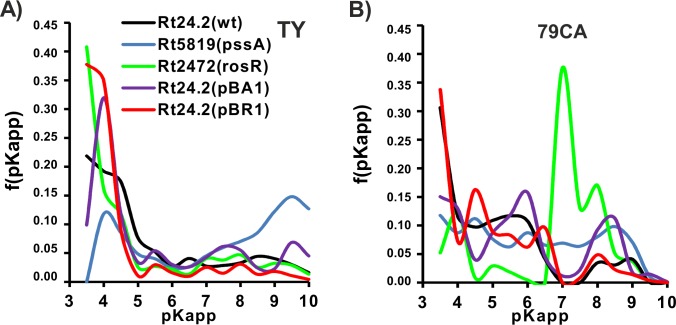
**Distribution functions [*f(pK*_*app*_*)*] of the apparent dissociation constants (*pK*_*app*_) of surface functional groups of Rt24.2, Rt5819, Rt2472, Rt24.2(pBA1), and Rt24.2(pBR1) strains grown in TY (A) and 79CA (B) media.**

To characterize the bacterial cell surface with respect to the presence of various functional groups comprising the source of surface electrical charge, we determined the distributions of their apparent dissociation constants [*f(pK*_*app*_*)*] in TY and 79CA media (Fig 4). An acidic apparent dissociation constant (*pK*_*app*_) describes the strength of an acid (or acidic group) in solution such that the lower the value, the stronger the acid. Distribution function of the apparent dissociation constants [*f(pK*_*app*_*)*] varies from 0 to 1, reflecting the input of groups characterized by particular *pK*_*app*_ to the total surface electrical charge.

We found that cell surface charge of the wild-type Rt24.2 and its derivatives was generated by different functional groups, depending on the culture medium and the strain analyzed. In TY medium, the surface charge of all analyzed strains was generated mainly by the dissociation of surface functional groups characterized by *pK*_*app*_<5.0 (e.g., pyruvic, carboxyl, and phosphoryl groups) (Fig 4A). For Rt24.2(pBA1) and Rt24.2(pBR1) strains, the participation of functional groups at *pK*_*app*_ 4.0 in total surface charge was higher than for Rt24.2. The lowest distribution function values were obtained for Rt2472 and Rt5819 mutants. For *pK*_*app*_>5.0, there were no significant differences between the investigated strains with respect to the distribution function values. An exception was the *pssA* mutant, where we identified an important role of functional groups with *pK*_*app*_>8.0 (e.g., amino acid groups from surface proteins).

In 79CA medium (Fig 4B), the distributions of the apparent dissociation constants were more varied between the tested bacterial strains. Strong acidic groups (*pK*_*app*_<5.0) played a significant role in surface charge generation, similarly to what was seen in TY medium. This was especially apparent for Rt24.2 and Rt24.2(pBR1) strains. Groups with *pK*_*app*_ in 4.5–6.5 range also significantly contributed to the surface charge of all strains, with the exception of Rt2472. For Rt2472, the most important groups on its cell surface were these with *pK*_*app*_ 6.5–9.0. This was not observed for other tested strains.

The average apparent dissociation constant (*pK*_*app*,*av*_) generally describes the chemical acid-base character of a surface. Similarly to the apparent dissociation constant (*pK*_*app*_), the lower the value, the more acidic the surface. In both media, *pK*_*app*,*av*_ values for the EPS-producing strains were very similar (Table 3). Among them, Rt24.2(pBR1) showed the most pronounced acidic character. The surface of Rt2472 cells was the least acidic in 79CA medium. Rt5819 surface had a weak acidic character in both media.

**Table 3:** Average apparent dissociation constants (*pK*_*app*,*av*_) for the wild-type *R*. *leguminosarum* bv. *trifolii* strain 24.2 and its derivatives grown in TY and 79CA media.

Medium	Rt24.2(wt)	Rt5819(*pssA*)	Rt2472(*rosR*)	Rt24.2(pBA1)	Rt24.2(pBR1)
TY	5.3 ± 0.2	7.5 ± 0.1	5.0 ± 0.2	5.7 ± 0.1	4.5 ± 0.1
79CA	4.9 ± 0.3	6.1 ± 0.2	6.7 ± 0.1	5.8 ± 0.2	4.9 ± 0.3

### Hydrophobicity/Hydrophilicity and SFE of *R*. *leguminosarum* bv. *trifolii* Cells

To determine the hydrophobic/hydrophilic properties of the studied strains grown in TY and 79CA media, CA measurements were performed using filters covered with air-dried bacteria and three liquids with differing polarities (water, diiodomethane, and formamide). In general, water CAs were moderately higher for all the analyzed rhizobial strains grown in 79CA compared with TY growth (Table 4). High values of water CA were measured for the wild type Rt24.2 and EPS-overproducing strains Rt24.2(pBR1) and Rt24.2(pBA1). The lowest values were measured for Rt5819 in both tested media, whereas the highest values were noted for Rt2472. This indicated that the cell surface of *pssA* mutant was more wettable while the surface of *rosR* mutant was less wettable than that of wild-type cells. CAs obtained using other liquids were much more diverse.

**Table 4:** Contact angle (CA) values for the wild-type *R*. *leguminosarum* bv. *trifolii* strain 24.2 and its derivatives.

Medium	Strain	CA [deg]*		
		ϴ_W_	ϴ_D_	ϴ_F_	
TY	Rt24.2 (wt)	67.4 ± 0.8	43.0 ± 1.7	58.0 ± 1.3
	Rt5819(*pssA*)	45.8 ± 1.1	51.0 ± 1.0	54.5 ± 2.6
	Rt2472(*rosR*)	77.3 ± 2.7	49.2 ± 0.7	58.3 ± 1.1	
	Rt24.2(pBA1)	65.9 ± 0.6	43.9 ± 0.7	63.9 ± 1.3		
	Rt24.2(pBR1)	74.6 ± 1.6	46.0 ± 1.7	55.8 ± 2.2		
79CA	Rt24.2 (wt)	77.1 ± 0.8	48.3 ± 0.3	48.9 ± 0.9
	Rt5819(*pssA*)	60.6 ± 1.3	37.5 ± 1.0	54.1 ± 1.6		
	Rt2472(*rosR*)	82.0 ± 1.5	51.7 ± 0.4	49.7 ± 0.6		
	Rt24.2(pBA1)	74.3 ± 0.3	49.0 ± 0.6	50.1 ± 1.0		
	Rt24.2(pBR1)	77.4 ± 1.4	48.8 ± 0.6	54.7 ± 1.2		

* CA values were determined using water (W), diiodomethane (D) and formamide (F).

All these values were used for the calculation of the total SFE and its components: electron acceptor component (γ^+^), electron donor component (γ^–^), polar acid-base component (AB), and apolar component (LW) (Table 5). These parameters enable a more precise characterization of bacterial cell surface properties. Total SFE values were similar in both media for all the studied strains. Among these, the lowest values were obtained for the *rosR* mutant, whereas the highest for the *pssA* mutant. LW was a dominant component of SFE and its values were very similar in all the analyzed strains. Both AB and γ^–^ component contributions were greatest in Rt5819 grown in TY and 79CA media and lower in Rt2472. Moreover, for Rt2472 strain, very high γ^+^ values were determined in both media, suggesting acidic character of the bacterial surface. However, this parameter was high in all EPS-producing strains.

**Table 5:** Surface free energy (SFE) and its components determined for the wild-type *R*. *leguminosarum* bv. *trifolii* strain 24.2 and its derivatives.

Medium	Strain	SFE [mJ m^-2^]*				
		γ+	γ-	AB	LW	Total
TY	Rt24.2 (wt)	1.9 ± 0.4	8.8 ± 1.0	8.1 ± 1.2	39.5 ± 0.9	47.6 ± 2.2
	Rt5819(*pssA*)	1.5 ± 0.6	32.0 ± 2.9	13.9 ± 3.2	35.0 ± 0.6	48.9 ± 3.8
	Rt2472(*rosR*)	3.7 ± 0.6	2.2 ± 1.3	5.7 ± 2.1	36.1 ± 0.4	41.8 ± 2.5
	Rt24.2(pBA1)	2.6 ± 0.2	13.9 ± 1.1	6.0 ± 1.2	39.0 ± 0.4	45.0 ± 1.6
	Rt24.2(pBR1)	3.7 ± 0.8	2.8 ± 1.1	6.5 ± 1.9	37.9 ± 1.0	44.3 ± 2.9
79CA	Rt24.2 (wt)	7.1 ± 0.5	0.5 ± 0.2	3.9 ± 0.9	36.6 ± 0.2	40.5 ± 1.1
	Rt5819(*pssA*)	1.7 ± 0.4	13.0 ± 1.7	9.3 ± 1.6	42.4 ± 0.5	51.7 ± 2.1
	Rt2472(*rosR*)	8.5 ± 0.5	0.0 ± 0.0	0.2 ± 1.5	34.6 ± 0.2	34.8 ± 1.7
	Rt24.2(pBA1)	6.2 ± 0.4	1.6 ± 0.2	6.3 ± 0.7	36.2 ± 0.5	42.5 ± 1.2
	Rt24.2(pBR1)	4.9 ± 0.5	1.4 ± 0.6	5.1 ± 1.4	36.3 ± 0.3	41.4 ± 1.7

* SFE components: γ^+^, electron-acceptor component; γ^–^, electron-donor component; AB, polar Lewis acid-base component; LW, apolar Lifshitz-van der Waals component.

In addition, the values of free energy of interaction between surfaces of two cells immersed in water (ΔG_BWB_) were calculated (Table 6). For most strains, ΔG_BWB_ values were below 0, indicating surface hydrophobicity [42]. Generally, ΔG_BWB_ values were more negative for cells grown in 79CA than TY. Values approaching 0 (least hydrophobic) and even positive (hydrophilic) were obtained for Rt5819 grown in 79CA and TY, respectively. The most negative ΔG_BWB_ value was noted for Rt2472 in TY medium.

**Table 6:** Free energy of interaction between cell surfaces of two bacterial cells immersed in water (ΔG_BWB_) calculated for the wild-type *R*. *leguminosarum* bv. *trifolii* strain 24.2 and its derivatives cultured in TY and 79CA media.

Strain	ΔG_BWB_ [mJ m^-2^]	
	TY	79CA
Rt24.2 (wt)	- 35.82	- 45.24
Rt5819(*pssA*)	+ 6.18	- 28.43
Rt2472(*rosR*)	- 48.19	- 46.05
Rt24.2(pBA1)	- 27.57	- 42.39
Rt24.2(pBR1)	- 46.65	- 47.54

### The Influence of pH on Cell Viability and Cell Surface Properties of *R*. *leguminosarum* bv. *trifolii*

**Fig 5:**
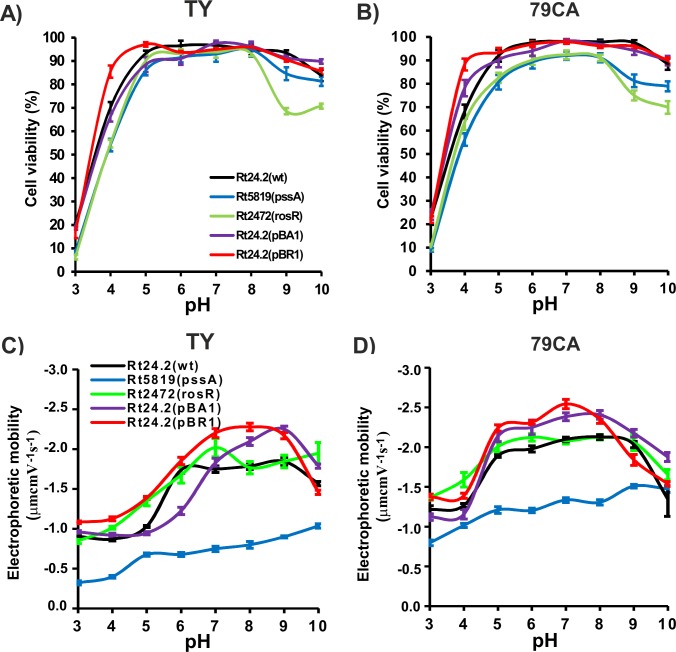
**The effect of pH (3–10) on the viability of Rt24.2, Rt5819, Rt2472, Rt24.2(pBA1), and Rt24.2(pBR1) strains grown in TY (A) and 79CA (B) media and on the electrophoretic mobility of bacterial cells in TY (C) and 79CA (D).**

pH is an important environmental factor, essentially affecting both bacterial survival and plant growth in the soil. Therefore, our further investigations focused on determining the influence of pH on the viability and surface properties of *R*. *leguminosarum* bv. *trifolii* cells. For this experiment, we used 24-h TY and 79CA cultures grown at pH 3 to 10. Bacterial survival profiles in TY and 79CA media over the tested pH range were similar, with pH values optimal for the survival of strains from 5 to 8 (Fig 5A and 5B). Within this pH range, >80% of bacterial population was comprised of live cells (depending on the strain tested). At higher pH values (9 and 10), the viability of Rt24.2, Rt24.2(pBR1), and Rt24.2(pBA1) was slightly decreased. These conditions more strongly affected the viability of both mutants, Rt5819 and Rt2472. Moreover, acidic conditions also strongly affected rhizobial viability. When pH was lowered from 5 to 3, a rapid decrease of live cell numbers was observed for all the examined strains (down to 20% in the case of Rt24.2, Rt24.2(pBR1), and Rt24.2(pBA1) strains; below 10% for Rt5819 and Rt2472 mutants). These data indicated that pH of the environment in which *R*. *leguminosarum* bv. *trifolii* cells reside is very important for their survival, and low pH (3–4) more drastically affects rhizobial viability than high pH (9–10). Furthermore, the mutants proved to be more sensitive to pH changes than wild type and this was especially apparent for the Rt2472 strain.

Next, we investigated the effect of pH on rhizobial strain EM. A strong impact of pH on EM was observed for both TY- and 79CA-grown bacteria (F_0.05;7;640_ = 851.93, p<0.001) (Fig 5C and 5D). Moreover, EM of individual strains was slightly higher when cells were grown in 79CA compared with TY cells (F_0.05;1;640_ = 2344.30, p<0.001), suggesting an additional effect of 79CA components on EM values. Generally, the EPS-producing strains had similar EM profiles in the analyzed pH range, although some differences between strains were observed. At pH 3 and 4, EM values were the lowest. They increased up to pH 7, remained on similar level in the pH range 7–9, and decreased at pH 10. Interestingly, the greatest differences in EM profiles from all the strains examined were found for Rt5819 mutant in both TY and 79CA media (Fig 5C and 5D). Rt5819 cells had much lower EM within the entire tested pH range, 3–10, than cells of the remaining strains. Moreover, highly acidic conditions (pH 3–4) additionally decreased EM of Rt5819 cells. All these data indicated that rhizobial strain type significantly influenced the EM values (F_0.05;4;640_ = 1447.80, p<0.001). Generally, for bacteria grown in both media, the EM was affected by the strain type and pH, and also by these two factors simultaneously (F_0.05;28;640_ = 22.80, p<0.001).

Furthermore, linear correlation was established between cell viability of the individual strains at different pH and their EM in given media (Table 7). We observed that the higher the number of live cells in a bacterial population, the more negative the EM value. The strongest correlations were noted for Rt2472 in 79CA and for Rt5819 in both media. The lowest, albeit still statistically significant, dependence of bacterial EM on cell viability was noted for Rt24.2(pBR1) strain in both media.

**Table 7:** Correlation coefficients (R) for EM values and the percentage of live cells in bacterial cultures of *R*. *leguminosarum* bv. *trifolii* strains.

Strain	The correlation coefficients (R)*	
	TY	79CA
Rt24.2 (wt)	-0.68	-0.66
Rt5819(*pssA*)	-0.74	-0.79
Rt2472(*rosR*)	-0.68	-0.82
Rt24.2(pBA1)	-0.58	-0.66
Rt24.2(pBR1)	-0.56	-0.58

* The data presented in the table are statistically significant (p < 0.05).

## Discussion

In this study, we provide evidence for a strong relationship between EPS production by *R*. *leguminosoarum* bv. *trifolii* strains and culture medium composition, and physicochemical surface properties of bacterial cells.

We established that 79CA medium was optimal for the growth and EPS production by the investigated strains (Figs 1 and 2). This can be explained by the fact that this medium contains more favored carbon and nitrogen sources than TY. Similar cell viability was observed in both media. Among the tested strains, the *pssA* and *rosR* mutants were characterized by slower growth and reduced viability.

The sign and magnitude of cell EM are frequently used as indicators of bacterial net surface electrical charge [34]. Therefore, we determined this parameter for the wild-type Rt24.2 strain and its derivatives that produced different amounts of EPS. We determined that the mean EM value was negative for all the analyzed strains in both TY and 79CA media. These data are consistent with other reports published for rhizobia [47,48].

The main finding of our work is that the absolute EM value of the *pssA* mutant with abolished EPS production was dramatically lower compared with EPS-producing strains under all conditions tested, indicating a significant role of this polymer in bacterial physicochemical properties. The EM values reflected the level of EPS synthesis and increased with an increasing amount of this polymer. This was observed for 24-h and 48-h cultures when live cells were highly prevalent in the bacterial population (>80% of all cells) (Fig 2). Our EM observations can be explained by a lower mobility of dead cells in an electric field compared with live ones, as established previously by us [35].

We noted that in 79CA medium, EM values were more negative than in TY medium for all the tested strains, including the EPS-deficient *pssA* mutant. This suggested an effect of medium composition. Although these media had the same pH, similar ionic strength, and electrolytic conductivity, they were different with respect to mono- and divalent ion concentrations. The TY medium was composed mainly of Ca^2+^ ions (~6 mM), whereas in 79CA medium, the total concentration of monovalent cations (Na^+^ and K^+^) was ~9 mM and the total concentration of divalent cations (Mg^2+^ and Ca^2+^) was 1 mM. In consequence, the negative bacterial surface charge was neutralized to a lesser extent in 79CA than in TY medium, resulting in greater cell mobility in the former. The effect of medium composition on bacterial cell EM values was also reported by other researchers [33,34].

Furthermore, to characterize bacterial cell surface properties in more detail, we have determined the EM distribution of individual strains in both media (Fig 3). Although the mean EM values for all strains were negative, the distribution of this parameter encompassed values higher than 0. This suggested a complex composition of the bacterial envelope. The positive EM values may be associated with the presence of RNH_3_^+^ groups [49]. The most apparent differences in EM profiles were observed for the Rt5819 strain, where the EM distribution range was significantly shifted toward positive values. This can be explained by the lack of EPS and CPS in *pssA* mutant envelope [11]. The dissociation of acidic functional groups present in these PSs is a source of the net negative surface charge of bacterial cells, and, in consequence, negative EM values [11,50]. Some authors pointed out the relationship between the synthesis of extracellular polymeric substances and bacterial EM [51,52].

Based on potentiometric titration experiments, we showed that the distribution of functional groups generating the surface electric charge was bacterial strain-specific and dependent on the composition of culture medium (Fig 4). In TY medium, surface electrical charge was generated mainly by the strong acidic groups with *pK*_*app*_<4.0 [53], most probably due to high Ca^2+^ concentration. Poortinga et al. [53] pointed out that PSs and phospholipids deliver the surface electric charge at pH ~3. From the strains tested, the Rt5819 mutant showed the lowest input of these groups to surface charge, but very high contribution of RNH_3_^+^ protein groups with *pK*_*app*_>9.0. In 79CA medium, where the synthesis of EPS by rhizobial strains was on the higher level than in TY, the role of the carboxyl groups in the surface charge generation was significant (*pK*_*app*_ 5.0–6.5) [54]. In the case of the Rt2472 strain, the participation of phosphoryl (from phospholipids) and amine (from proteins) groups (*pK*_*app*_ 7.0–9.0) was essential in total surface charge [53,54]. In addition, we found that bacterial cell surface became more acidic with increasing amounts of synthesized EPS, as described by the average apparent dissociation constant *pK*_*app*,*av*_ (Table 3).

Regardless of the acidic character of bacterial surface and the net negative electrical charge, the envelopes of air-dried tested bacteria were hydrophobic, as assessed by the determined ΔG_BWB_ values [46] (Tables 4 and 6). Generally, cell hydrophobicity between the analyzed genetically modified strains was different. Cell surface hydrophobicity is underpinned by a complex composition of both the envelope and extracellular matrix surrounding the cell. The matrix is composed of PSs (40–95%), proteins (1–60%), and lipids (1–10%) [55,56]. Its hydrophilic nature is associated with the presence of hydrated PSs, proteins, and DNA molecules. However, More et al. [55] suggested that polysaccharide-linked methyl and acetyl groups present in the matrix, as well as lipids and their derivatives, were responsible for the hydrophobic character of the cell. In addition, the EPS matrix may contain lipids and their derivatives [55]. Moreover, the presence of mono- and divalent cations in culture media leads to increased hydrophobicity of surfaces. Cell surfaces of all strains were more hydrophobic in the mineral nutrient-rich 79CA medium than in TY medium. Similar results were obtained by Mehmannavaz et al. [57] who compared bacteria grown in TY and YEMP media. PSs may be more hydrophilic or hydrophobic depending on their three-dimensional conformation [44]. Negative ΔG_BWB_ value indicates attraction between cells immersed in water [46], which can be important during bacterial behavior both in a free-living stage and during early symbiotic interaction with the host plants. In both media (TY and 79CA), the least hydrophobic surface was that of Rt5819 strain. The higher the cell hydrophobicity, the better the ability to adhere to different surfaces [58], similarly to what was observed by us previously in an investigation of rhizobial attachment to biotic and abiotic surfaces and biofilm formation [10].

SFE provides general information about the physicochemical properties of surfaces (Table 5). It is the sum of Lewis acid-base (AB) and the Lifshitz- van der Waals (LW) components. The first component reflects the electro-acceptor (acid) and electro-donor (base) character of the surface. LW component covers the London dispersion, Keesom orientation, and Debye induction forces [44,56]. LW component values obtained by us were in the range of 35–42.4 mJ•m^-2^, depending on the strain and culture medium. For biological molecules and cells, these values range from 20 mJ•m^-2^, for hydrocarbons and lipids, to 42 mJ•m^-2^, for proteins or PSs [45]. This component dominated the total SFE value. In both media, the strains characterized by high negative EM and EPS production exhibited an electro-acceptor (acidic) character (γ^+^) of the cell surface [44].

Further, pH also strongly affected bacterial viability and EM values of the rhizobial cells (Fig 5). The optimal conditions for bacterial survival were from pH 5 to 9, consistently with the literature [59,60]. At low pH, the availability of Ca^2+^ cations, which are important for outer membrane stability, is low [59, 60]. Generally, *R*. *leguminosarum* bv. *trifolii* cannot grow in the soil at pH <4.7 [61,62]. Some strains tolerant to acidity have developed resistance mechanisms such as increased EPS production [61–63]. However, the strains investigated by us produce most EPS within pH range optimal for their growth [10]. Thus, the low EM values observed by us in strong acidic conditions resulted from both, diminished amount of EPS and high number of dead cells (Table 7) in bacterial suspension [35]. EM increase at pH 4–5 was a consequence of increasing EPS production, higher number of live cells in a population, and increasing dissociation of the weaker acidic groups [10,53,54]. Similarly, other reports also showed that EPS production is pH-dependent [63–65].

Moreover, we observed that in all pH ranges in TY and 79CA media, EM values of bacteria that produced acidic EPS, rich in carboxyl groups [53], were more negative than that of *pssA* mutant. In addition, EM of this mutant was not significantly affected by pH. Changes of rhizobial surface properties at different pH values observed by us might play an important role during the first step of symbiotic interaction between a bacterium and the host plant [8,11]. It is known that EPS production facilitates the adhesion of bacterial cells to different surfaces [10]. EPS interacts with the surfaces by noncovalent bonds, i.e., electrostatic attraction and hydrogen bonds, with hydrophobic properties of the bacterial cell envelope playing an important role [56].

## Conclusions

The production of EPS by rhizobia strongly influences their cell surface properties. We observed that the higher the amount of EPS produced, the more negative the EM value, and simultaneously, the more significant role of carboxyl group as a source of surface electrical charge. However, employment of EM as a predictor of EPS amounts should comprise considerations of the state of bacterial population (live/dead cell ratio), and the qualitative and quantitative composition of the dispersing medium. The measured EM values were strain-specific. The *pssA* mutant, in which EPS synthesis was totally abolished, had the lowest absolute EM value from the tested strains. Furthermore, the presence of EPS increased the contribution of electro-acceptor parameter of the polar component of SFE. We found that the hydrophobicity of air-dried rhizobial cell envelopes increased with increasing EPS amount, explaining enhanced cell aggregation in aqueous media. In addition, we observed a significant influence of pH on both bacterial survival and EM values. pH-dependent changes in EM were strain-dependent. In contrast with the tested EPS-producing strains, EM of the *pssA* mutant was not significantly affected by pH.
